# A reference genome, mitochondrial genome and associated transcriptomes for the critically endangered swift parrot ( *Lathamus discolor*)

**DOI:** 10.12688/f1000research.144352.1

**Published:** 2024-04-04

**Authors:** Luke W. Silver, Dejan Stojanovic, Katherine A. Farquharson, Lauren Alexander, Emma Peel, Katherine Belov, Carolyn J. Hogg

**Affiliations:** 1 School of Life and Environmental Sciences, The University of Sydney, Sydney, New South Wales, 2006, Australia; 2 Fenner School of Environment and Society, Australian National University, Acton, Australian Capital Territory, 2601, Australia; 3 Australian Research Council Centre of Excellence for Innovations in Peptide and Protein Science,, The University of Sydney, Sydney, New South Wales, 2006, Australia

**Keywords:** Genome assembly, reference genome, transcriptome, Aves, mitogenome

## Abstract

The swift parrot ( *Lathamus discolor*) is a Critically Endangered migratory parrot that breeds in Tasmania and winters on the Australian mainland. Here we provide a reference genome assembly for the swift parrot. We sequence PacBio HiFi reads to create a high-quality reference assembly and identify a complete mitochondrial sequence. We also generate a reference transcriptome from five organs to inform genome annotation. The genome was 1.24 Gb in length and consisted of 847 contigs with a contig N50 of 18.97 Gb and L50 of 20 contigs. This study provides an annotated reference assembly and transcriptomic resources for the swift parrot to assist in future conservation genomic research.

## Introduction

The swift parrot ( *Lathamus discolor*) is a migratory parrot that breeds on the island of Tasmania, Australia and winters on mainland Australia ( Kennedy & Tzaros, 2005; MacNally & Horrocks, 2000; Saunders & Heinsohn, 2008). The swift parrot is Critically Endangered ( BirdLife International, 2018) due to the combined effects of logging of its important breeding habitat ( Webb *et al.*, 2019) and the impacts of an introduced predator ( Heinsohn *et al.*, 2015). Population viability analysis has shown that the already small population of only a few hundred swift parrots ( Olah *et al.*, 2021) is likely to rapidly decline over coming generations ( Heinsohn *et al.*, 2015; Owens *et al.*, 2023) Although the species has already been subject to population genetic study ( Olah *et al.*, 2021; Stojanovic *et al.*, 2018), there remain outstanding questions about multiple aspects of the species’ genetic ecology. For example, like other parrots with small population sizes ( Morrison *et al.*, 2020), understanding the genetic basis of immune competence is critical for managing demographic impacts of disease in swift parrots ( Saunders & Tzaros, 2011). To facilitate detailed genomic research on this species, we sequenced DNA with PacBio long reads to generate a high-quality reference assembly and sequenced RNA from five tissues to provide transcriptomic resources and assist in genome annotation for the swift parrot.

## Methods

### Sample collection and DNA/RNA extraction

A single captive bred female swift parrot died as a result of liver infection. Tissue samples were dissected and flash frozen at -80°C or preserved in RNAlater before being frozen at -80°C. High molecular weight (HMW) DNA was then extracted from heart and kidney tissue using the Nanobind Tissue Big DNA Kit v1.0 (Circulomics: SKU 102-302-100) using the standard protocol. A Qubit fluorometer was used to assess the concentration of DNA with the Qubit dsDNA BR assay kit (Thermo Fisher Scientific). Total RNA was extracted from gonad, spleen, liver, heart and kidney using the RNeasy Plus Mini Kit (Qiagen: 74134) with RNAse-free DNAse I set (Qiagen: EN0521) using the standard protocol. RNA quality was determined using the NanoDrop (Thermo Fisher Scientific) and RNA integrity (RIN) score determined using the Bioanalyzer RNA nano 6000 kit (Agilent 2100: 5067-1511).

### Library construction and sequencing

HMW DNA was sent for Pacific Biosciences High Fidelity (PacBio HiFi) library preparation with the SMRTbell Express Template Prep Kit 2.0 (Pacific Biosciences: 101-853-100) and sequencing on one single molecule real-time (SMRT) cell of the PacBio Sequel II at the Australian Genome Research Facility (St Lucia, Australia). Total RNA from the heart, gonad, kidney, liver and spleen was sequenced as 100 bp paired-end (PE) reads using an Illumina Novaseq 6000 with Illumina Stranded mRNA library preparation at the Ramaciotti Centre for Genomics (University of New South Wales, Kensington, Australia).

### Genome assembly

The genome assembly was conducted on the Galaxy Australia public server usegalaxy.org.au ( Afgan *et al.*, 2016) running the Genome assembly with ‘hifiasm’ (RRID:SCR_021069) ( Cheng *et al.*, 2022) on Galaxy Australia workflow v2.1 ( Price & Farquharson, 2022). Briefly, Picard ( http://broad institute.github.io/picard) (Galaxy version 2.18.2.2; RRID:SCR_006525) *SamToFastq*, samtools ( Danecek *et al.*, 2021; Li *et al.*, 2009) (Galaxy version 2.0.3; RRID:SCR_002105) *flagstat* and fastQC ( https://www.bioinformatics.babraham.ac.uk/projects/fastqc/) (Galaxy version 0.72; RRID:SCR_014583) was used to convert BAM files to FASTQ and quality check the reads for input to Hifiasm ( Cheng *et al.*, 2022). Hifiasm (Galaxy version 2.1) was run on Galaxy Australia to assembly the genome. Basic genome assembly statistics were calculated with the stats.sh script in BBMap ( sourceforge.net/projects/bbmap/) (RRID:SCR_016965). Genome completeness was determined using Benchmarking Universal Single-Copy Orthologues (BUSCO; RRID:SCR_015008) v5.4.6 ( Simao *et al.*, 2015) with the vertebrata_odb10 (n = 3354) and aves_odb10 (n= 8338) lineage on Galaxy Australia. Repetitive elements of the genome were identified, classified and masked using a Pawsey Supercomputing Centre Nimbus cloud machine (256GB RAM, 64 vCPU, 3 TB storage) by building a database using RepeatModeler v2.0.1 (RRID:SCR_015027) ( Flynn *et al.*, 2020); repeats were then masked using RepeatMasker v4.0.9 (RRID:SCR_012954) ( Smit *et al.*, 2013-2015) with the *-nolow* parameter to avoid masking low complexity repeats.

### Mitochondrial assembly

The mitochondrial genome was identified from the reference genome assembly using MitoHiFi v2 ( Allio *et al.*, 2020; Uliano-Silva *et al.*, 2023) and visualised using Proksee ( Grant *et al.*, 2023). MitoHifi identified the most taxonomically closely related publicly available mitochondrial genome, the thick-billed parrot ( *Rhynchopsitta pachyrhyncha*) (NCBI reference sequence OR209192.1), used to search for the swift parrot mitochondrial genome.

### Transcriptome assembly

Transcriptome assembly was performed on the University of Sydney High Performance Computer, Artemis. Raw transcriptome reads were quality assessed pre and post trimming with FastQC v0.11.8 (RRID:SCR_014583). Trimmomatic v0.39 (RRID:SCR_011848) ( Bolger *et al.*, 2014) with the parameters SLIDINGWINDOW:4:5, LEADING:5, TRAILING:5 and MINLEN:25 and ILLUMINACLIP:2:30:10 with the TruSeq3-PE adapters was used to quality trim reads. The repeat masked genome was indexed and trimmed reads aligned using the *-dta* parameter with hisat2 v2.1.0 (RRID:SCR_015530) ( Kim *et al.*, 2019). Resulting sam files with converted to bam format and sorted using samtools v1.9 ( Danecek *et al.*, 2021). Stringtie v2.1.6 (RRID:SCR_016323) ( Pertea *et al.*, 2015) was used to generate a GTF for each transcriptome. Stringtie v2.1.6 with the *-merge* parameter merged transcripts into a global transcriptome retaining only transcripts with an FPKM > 0.1 and length > 30. CPC2 v2019-11-19 ( Kang *et al.*, 2017) was used to predict coding potential and only transcripts predicted to be coding were retained. Transdecoder v2.0.1 ( https://github.com/TransDecoder/TransDecoder) (RRID:SCR_017647) was used to predict open reading frames in the global transcriptome with a minimum transcript length of 20. Transcriptome completeness was assessed using BUSCO v5.4.6 ( Simao *et al.*, 2015) with the vertebrata_odb10 (n = 3354) and aves_odb10 (n = 8338) lineage on Galaxy Australia.

### Genome annotation

Genome annotation was performed using FGENESH++ v7.2.2 (Softberry; RRID:SCR_018928 ( Solovyev *et al.*, 2006)) using the longest open reading frame as predicted from the global transcriptome, non-mammalian settings and optimised parameters supplied with the American crow ( *Corvus brachyrhynchos*) gene finding matrix. BUSCO v5.4.6 ( Simao *et al.*, 2015) in protein mode was run on Galaxy Australia to assess the completeness of the annotation with the vertebrata_odb10 (n = 3354) and aves_odb10 (n = 8338) lineage. The ‘genestats’ script ( https://github.com/darencard/GenomeAnnotation) was used to obtain the average number of exons and introns and the average exon and intron length.

## Results

### Genome assembly

**Figure 1.**
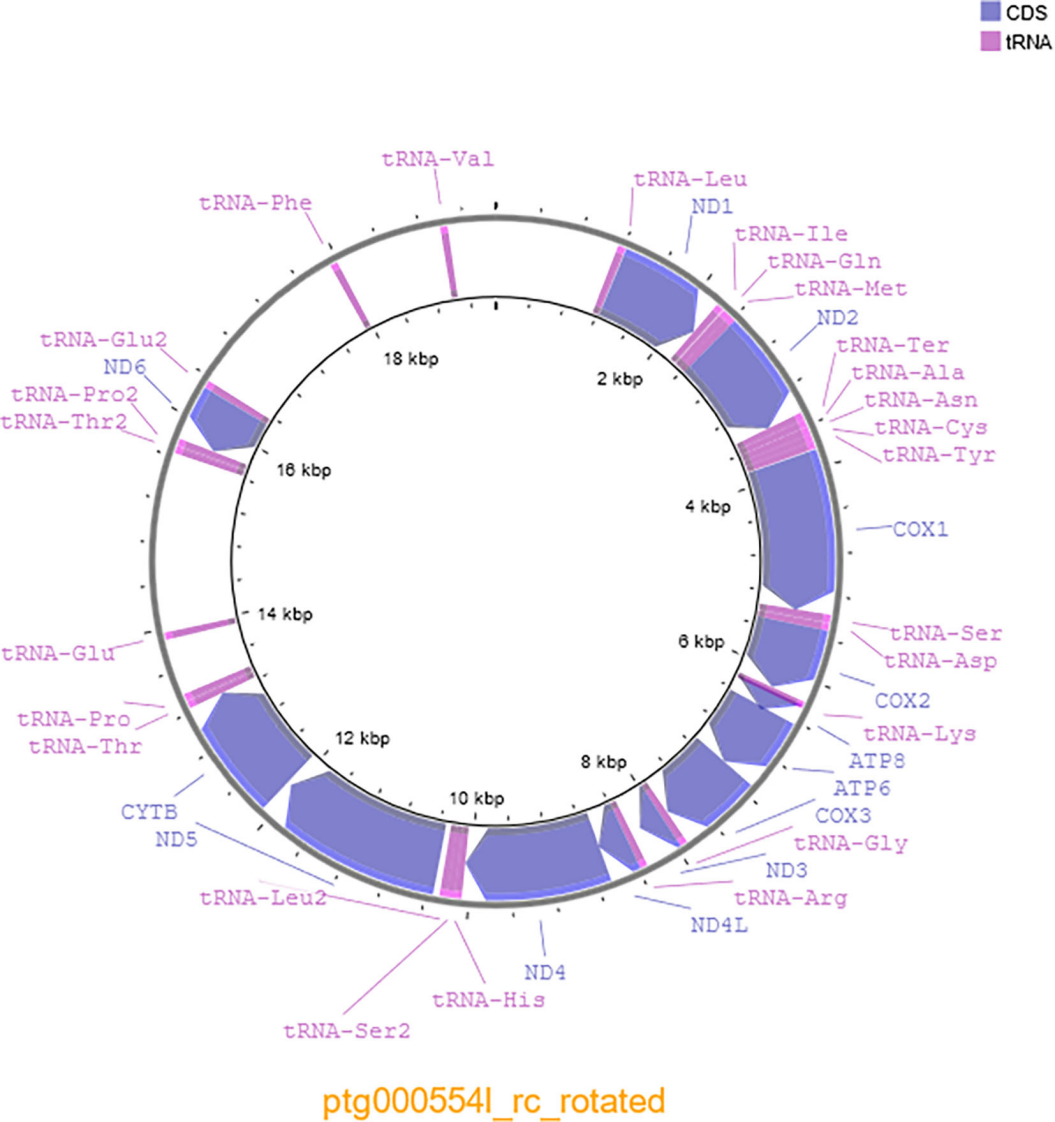
Mitochondrial genome of the swift parrot ( *Lathamus discolor)* generated with Proksee ( Grant *et al.*, 2023).

Genome assembly using Hifiasm with PacBio HiFi data from a single SMRT cell resulted in a coverage of 28.7x and a genome of 1.24 Gb in size consisting of 847 contigs with a contig N50 of 18.97 Mb and L50 of 20 contigs. The genome assembly was also highly complete with 97.0% of aves_odb10 complete BUSCOs identified ( Table 1). The mitochondrial genome was 19,498 bp long and contained 38 genes, including 25 tRNAs and 13 protein coding genes, with a GC percentage of 44.59% ( Figure 1).

**Table 1.** Genome assembly statistics of the swift parrot ( *Lathamus discolor*) with statistics calculated with the stats.sh script as part of the BBMap software ( https://sourceforge.net/projects/bbmap/) and BUSCO ( Simao *et al.*, 2015) completeness, calculated with both the vertebrata_obd10 and aves_obd10 lineages.

Metric	
Assembly size (Gb)	1.24
Number of contigs	847
Contig N50 (Mb)	18.97
Contig N90 (Mb)	2.46
Contig L50	20
Contig L90	83
Longest contig (Mb)	78.39
GC content (%)	42.8
Complete vertebrata_odb10 BUSCOs	96.3% (Single copy: 94.7%, Duplicated: 1.6%)
Fragmented vertebrata_odb10 BUSCOs	1.0%
Missing vertebrata_odb10 BUSCOs	2.7%
Complete aves_odb10 BUSCOs	97.0% (Single copy: 96.1%, Duplicated: 0.9%)
Fragmented aves_odb10 BUSCOs	0.5%
Missing aves_odb10 BUSCOs	2.5%

### Transcriptome assembly and genome annotation

Trimming retained greater than 99.95% of raw reads which were then aligned to the repeat-masked reference genome. Individual tissue transcriptomes had variable mapping rates from 31.04% for heart tissue to 82.76% for gonad tissue (kidney: 62.26%, liver: 78.84%, spleen: 73.60%). The alignment rate for the heart tissue was low so we excluded heart transcripts from downstream analysis. The poor performance of the heart tissue is potentially due to the comparatively lower concentration of RNA in the heart tissue extraction (35.2 ng/μl) compared to the other 4 tissues (average = 1243 ng/μl [SD: 481]) and the heart tissue was not stored in RNAlater. After using stringtie *-merge* to generate a global transcriptome and filtering on coding potential and open reading frames with CPC2 and transdecoder, respectively, 14,045 longest open reading frame transcripts were used as mRNA evidence to guide genome annotation. The global transcriptome had 90.8% complete aves_odb10 BUSCOs ( Table 2). A total of 27,867 genes were predicted from genome annotation, higher than the predicted 15,000-16,000 genes in birds ( Zhang *et al.*, 2014). The annotation contained 78.1% complete aves_odb10 BUSCOs ( Table 2). Repetitive elements comprised 17.25% of the genome, mainly consisting of long interspersed elements (LINEs), comparable with other bird genomes ( Zhang *et al.*, 2014) ( Table 3).

**Table 2.** Statistics of the global transcriptome and annotation of the swift parrot ( *Lathamus discolor*) including BUSCO ( Simao *et al.*, 2015) completeness, calculated with both the vertebrata_obd10 and aves_obd10 lineages and average exon length.

Metrics	
Global Transcriptome	
Complete vertebrata_odb10 BUSCOs	94.8% (Single copy: 33.5%, Duplicated: 61.3%)
Fragmented vertebrata_odb10 BUSCOs	1.1%
Missing vertebrata_odb10 BUSCOs	4.1%
Complete aves_odb10 BUSCOs	90.8% (Single copy: 32.8%, Duplicated 58.0%)
Fragmented aves_odb10 BUSCOs	1.1%
Missing aves_odb10 BUSCOs	8.1%
Annotation	
Complete vertebrata_odb10 BUSCOs	72.5% (Single copy: 70.4%, Duplicated: 2.1%)
Fragmented vertebrata_odb10 BUSCOs	10.6%
Missing vertebrata_odb10 BUSCOs	16.9%
Complete aves_odb10 BUSCOs	78.1% (Single copy: 77.2%, Duplicated: 0.9%)
Fragmented aves_odb10 BUSCOs	5.4%
Missing aves_odb10 BUSCOs	16.5%
Average number of exons per gene	7.84
Average number of introns per gene	6.84
Average exon length (bp)	2368
Average intron length (bp)	22346

**Table 3.** Classification of repeat elements of the swift parrot ( *Lathamus discolor*) genome assembly as generated by the repeatmasker software ( Smit *et al.*, 2013-2015).

Repeat element	Number of elements	% of sequence
SINEs	2917	0.03
MIRs	1302	0.01
LINES	252190	8.11
LINE1	831	0.01
LINE2	528	0
L3/CR1	250524	8.08
LTR elements	26360	1.38
ERVL	10824	0.42
ERV Class I	9460	0.56
ERV Class II	4642	0.23
DNA elements	4580	0.04
*hAT*- *Charlie*	334	0
Unclassified	105511	7.69
Total interspersed repeats		17.25
Small RNA	2275	0.09

### Ethical considerations

The sample used for genome and transcriptome sequencing was obtained from an individual who died of natural causes.

## Data Availability

The raw PacBio HiFi and transcriptome data are publicly available through the Bioplatforms Australia Threatened Species Initiative: https://data.bioplatforms.com/organization/threatened-species . The assembled genome, global transcriptome and annotation generated in this study are available on Amazon Web Services Australasian Genomes Open Data Store: https://awgg-lab.github.io/australasiangenomes/genomes.html. Raw genome and transcriptome sequences are also available from NCBI’s Short Read Archive (SRA) accession numbers SRR26186073 to SRR26186078 ( Silver *et al.*, 2023). And the assembled genome from NCBI’s Assembly database, BioProject: PRJNA1021263 ( Silver *et al.*, 2023). Figshare: Author Checklist - ARRIVE.pdf, https://doi.org/10.6084/m9.figshare.25396294. Data are available under the terms of the Creative Commons Zero “No rights reserved” data waiver (CC0 1.0 Public domain dedication).
