# Supplementary material for: A reference genome, mitochondrial genome and associated transcriptomes for the critically endangered swift parrot ( Lathamus discolor)
Source: F1000Res. 2024 Aug 27;13:251. Originally published 2024 Apr 4. [Version 2] doi: 10.12688/f1000research.144352.2 (PMC11411239; doi:10.12688/f1000research.144352.2)
Supplement: Peer review history

# Reviewer report: Reviewer response for version 2

**DOI:** 10.5256/f1000research.170611.r318594

**Published:** 2024-09-18

**Authors:** Joan Ferrer Obiol

**Affiliations:** 1 University of Milan, Milan, Italy; 2 Universitat de Barcelona (UB), Barcelona, Spain

Silver et al. present a highly-contiguous reference genome for the Critically Endangered Swift parrot. The methods used are sound but I have a few comments and suggestions:

 Abstract: 

 Line 5: there is a mistake on the units of the N50 and should probably be changed to Mb rather than Gb.

 Introduction:

 Line 4: I suggest changing "its important breeding habitat" to something more informative about the type of habitat that the species occupies.

 Line 8: I suggest changing genetic ecology by a more appropriate concept such as ecological genetics.

 Line 11: Consider changing "we sequenced DNA with PacBio long reads to generate a draft reference assembly" to something like "we produced a draft genome assembly for the species using PacBio long reads"

 Methods:

 Transcriptome assembly:

 I would encourage the authors to make it clear to the reader why they decided to remove the non-coding transcripts from the transcriptome and why they decided to not use them for the annotation. The important role of long non-coding RNAs in evolution is increasingly being demonstrated (e.g. Toomey et al. 2018; Mattick et al. 2023) and thus it seems strange to ignore them.

 Results:

 Genome assembly:

 Regarding the mitogenome, I would suggest mentioning that the mitogenome was circularized. Also, I would consider annotating the two rRNA genes and the control region in Proksee.

 Transcriptome assembly and genome annotation:

 Given the low BUSCO completeness of the annotation, despite the relatively high completeness of the transcriptome, I would encourage the authors to use an alternative genome annotation pipeline that makes a better use of the RNASeq data such as BRAKER3.

 In addition, given the high number of genes obtained in the annotation, I would consider doing some kind of filtering of the raw annotation, e.g. using gFACs (Ref 3) to remove potentially missannotated genes.

**Literature cited in the review:**

 Mattick, J.et.al. 2023 (Ref 1)

 Toomey, M. B.et.al., 2018 (Ref 2)

 Caballero, M.et.al., 2019 (Ref 3)

Are the datasets clearly presented in a usable and accessible format, and the assembly and annotation available in an appropriate subject-specific repository?

Yes

Are sufficient details of the sequencing and extraction, software used, and materials provided to allow replication by others?

Yes

Are the rationale for sequencing the genome and the species significance clearly described?

Yes

Are the protocols appropriate and is the work technically sound?

Partly

Reviewer Expertise:

Population genomics, phylogenomics, ecological genomics and some experience in genome assembly

I confirm that I have read this submission and believe that I have an appropriate level of expertise to confirm that it is of an acceptable scientific standard.

## References

[1] : Long non-coding RNAs: definitions, functions, challenges and recommendations. *Nat Rev Mol Cell Biol* .2023;24(6) : 10.1038/s41580-022-00566-8 430-447 10.1038/s41580-022-00566-8 36596869 PMC10213152

[2] : A non-coding region near Follistatin controls head colour polymorphism in the Gouldian finch. *Proc Biol Sci* .2018;285(1888) : 10.1098/rspb.2018.1788 10.1098/rspb.2018.1788 30282656 PMC6191701

[3] : gFACs: Gene Filtering, Analysis, and Conversion to Unify Genome Annotations Across Alignment and Gene Prediction Frameworks. *Genomics Proteomics Bioinformatics* .2019;17(3) : 10.1016/j.gpb.2019.04.002 305-310 10.1016/j.gpb.2019.04.002 31437583 PMC6818179

# Reviewer report: Reviewer response for version 2

**DOI:** 10.5256/f1000research.170611.r317694

**Published:** 2024-09-10

**Authors:** Phred Benham

**Affiliations:** 1 University of Massachusetts Amherst Department of Biology (Ringgold ID: 117236), Amherst, Massachusetts, USA

The authors have addressed my comments and those of the other reviewer. I have no further comments to add.

Are the datasets clearly presented in a usable and accessible format, and the assembly and annotation available in an appropriate subject-specific repository?

Yes

Are sufficient details of the sequencing and extraction, software used, and materials provided to allow replication by others?

Yes

Are the rationale for sequencing the genome and the species significance clearly described?

Yes

Are the protocols appropriate and is the work technically sound?

Yes

Reviewer Expertise:

genomics, ornithology, evolution

I confirm that I have read this submission and believe that I have an appropriate level of expertise to confirm that it is of an acceptable scientific standard.

# Reviewer report: Reviewer response for version 1

**DOI:** 10.5256/f1000research.158132.r296521

**Published:** 2024-07-13

**Authors:** Phred Benham

**Affiliations:** 1 University of Massachusetts Amherst Department of Biology (Ringgold ID: 117236), Amherst, Massachusetts, USA

This manuscript describes a de novo assembly of the critically endangered swift parrot.

 Generally the sequencing and assembly methods reflect current standards and they produce a highly contiguous, contig-level assembly for this species that will be of value for various conservation genomics and other molecular ecology questions. I have only a few minor comments.

 Is there a reason you did not attempt to produce a scaffolded assembly?

 If there is space it would be nice to have a figure(s) showing the parrot, distribution, etc.

 I agree with the other reviewer that this assembly is larger than the typical avian mito-genome and worth confirming there is not spurious sequence included.

 Was a voucher specimen preserved of the parrot that died?  

 The last sentence of the 'mitochondrial assembly' section is not clearly written, please revise.

 Resulting sam files 'were' not 'with' converted...

 Why did you use crow for the gene finding matrix. Were there not other parrots or something more closely related?

Are the datasets clearly presented in a usable and accessible format, and the assembly and annotation available in an appropriate subject-specific repository?

Yes

Are sufficient details of the sequencing and extraction, software used, and materials provided to allow replication by others?

Yes

Are the rationale for sequencing the genome and the species significance clearly described?

Yes

Are the protocols appropriate and is the work technically sound?

Yes

Reviewer Expertise:

genomics, ornithology, evolution

I confirm that I have read this submission and believe that I have an appropriate level of expertise to confirm that it is of an acceptable scientific standard, however I have significant reservations, as outlined above.

# Response

**Published:** 2024-08-19

**Authors:** Luke Silver

**Affiliations:** The University of Sydney, Sydney, New South Wales, Australia

We did not attempt a scaffolded assembly as for conservation and population genetics work we have found a long read assembly to be sufficient, additionally after we produced our long read assembly a chromosome level scaffolded assembly was released by VGP.

 In lieu of adding in an additional figure showing the distribution of the swift parrot, we have added some additional detail as to the regions where swift parrots breed and migrate to

 “The swift parrot ( *Lathamus discolor*) is a migratory parrot that breeds on the eastern seaboard of the island of Tasmania, Australia and winters on southeastern mainland Australia”

 As requested by the other reviewer I have ensured the mitochondrial information included in the manuscript and online is correct and updated to reflect this.

 The individual used for the genome and transcriptome will be accessioned into a museum collection by the end of 2024, when this has been completed the BioProject information on NCBI will be updated to provide the accession number

 I have rephrased this to make sense. “…genome as, the thick-billed parrot ( Rhynchopsitta pachyrhyncha) (NCBI reference sequence OR209192.1). The mitochondrial reference sequence for the thick-billed parrot was then, used…”

 I have changed with to were

*The crow is the most closely related species with a matrix provided by FGENESH, I have edited the methods to reflect this. “…gene finding matrix, which is the closest related species with a gene finding matrix provided by FGENESH++”*

# Reviewer report: Reviewer response for version 1

**DOI:** 10.5256/f1000research.158132.r274647

**Published:** 2024-06-04

**Authors:** Charles Feigin

**Affiliations:** 1 Department of Environment and Genetics, La Trobe University (Ringgold ID: 2080), Melbourne, Victoria, Australia

Here, the authors present a short report detailing the production of nuclear and mitochondrial genome assemblies for the swift parrot *Lathamus discolor*, together with annotations supported by multi-tissue transcriptomes. The swift parrot is a Critically Endangered species and the target of conservation efforts, making these new data and resources valuable. Overall, the report is well written, and I appreciated the very detailed methods sections with parameters spelled out. I had one notable comment related to data availability and sequence length of the mitochondrial genome assembly. Further comments are fairly minor given the straightforward nature of the genome report.

**Data availability and length discrepancy of the mitochondrial genome assembly **- While other data in the paper has been made readily available and are associated with the BioProject on NCBI (or on FigShare or the AWS database), it is unclear whether the mitochondrial assembly has been made available.

 I found that the genome assembly itself on NCBI contains a contig that has been labelled as the mitochondrial genome, JAXCIO010000845.1. However, the length of this sequence (17,265 bp) differs substantially from the mitochondrial genome length reported in the paper (19,498 bp). Is this the same mito assembly described in the paper, or was this contig produced during the *de novo *assembly of the nuclear genome? I note that the ~17 kbp sequence included in your genome file is more typical for the length of vertebrate mitochondria and is much closer in size to the related parrot you indicated in the paper ( *Rhynchopsitta pachyrhyncha*, OR209192.1) than the ~19kb produced by MitoHifi v2 and reported in-text. I request the following:

1. If the mitochondrial contig JAXCIO010000845.1 included in the assembly file is the same mitochondrial assembly described in the report, then the length discrepancy between this contig and what is reported in the paper needs to be addressed.
2. If JAXCIO010000845.1 is not the same as the mito assembly reported in the paper, then 1) your *de novo* ~19kb mitochondrial genome needs to be deposited into Genbank and associated with your BioProject and 2) the length discrepancy between these two should still be addressed,

**"and the impacts of an introduced predator" -  **it is probably worth spelling out what the predator is.

**"to generate a high-quality reference assembly" -** . Chromosome-level *de novo* genome assemblies have become standard, even for vertebrates, and are very slowly starting to be superseded by T2T assemblies. Given current standards and the recent VGP *L. discolor* reference genome, the assembly presented here would best be described a draft assembly throughout.

**Genome assembly methods** - What Hifiasm parameters were used? If default that's fine, just note it.

**Table 3 "DNA elements"** - The label "DNA elements" is default for RepeatMasker but its a fairly vague label for these elements. It would be helpful to rephrase this as "DNA Transposons" to be more specific/clear about what they are.

Are the datasets clearly presented in a usable and accessible format, and the assembly and annotation available in an appropriate subject-specific repository?

No

Are sufficient details of the sequencing and extraction, software used, and materials provided to allow replication by others?

Yes

Are the rationale for sequencing the genome and the species significance clearly described?

Yes

Are the protocols appropriate and is the work technically sound?

Yes

Reviewer Expertise:

Evolutionary Biology, Developmental Biology, Genomics, Marsupials

I confirm that I have read this submission and believe that I have an appropriate level of expertise to confirm that it is of an acceptable scientific standard, however I have significant reservations, as outlined above.

# Response

**Published:** 2024-08-19

**Authors:** Luke Silver

**Affiliations:** The University of Sydney, Sydney, New South Wales, Australia

The mitochondrial contig listed on NCBI is the correct mitochondrial assembly, it appears that MitoHifi annotates the entire contig which was identified to contain mitochondrial genome even if it is much larger than the expected size.

 I had replaced the identified contig with only the portion of sequence which represented the mito genome in the assembly file, however not the data which was used to annotate and produce figure 1.

 I have edited and updated the manuscript to reflect the changes. *“The mitochondrial reference sequence for the thick-billed parrot was then, used to search for the swift parrot mitochondrial genome. The identified mitochondrial sequence was then added to the genome assembly and annotated using MITOS v 2.1.7 (Donath et al., 2019) and visualised using Proksee ( Grant et al., 2023).”*

 I have edited the sentence to name the predator *“… and the impacts of an introduced predator, the sugar glider (Petaurus breviceps)*

 I have replaced high-quality with draft when referring to the genome assembly throughout

 I have stated that hifiasm was run with default parameters, “ *Hifiasm, with default parameters, was run on Galaxy Australia…”*

 I have relabelled DNA elements as DNA transposons in Table 3
